# Defining and Assessing Empathic Communication in Patient Portal Secure Messages: Adapted Coding Framework Development Study

**DOI:** 10.2196/87195

**Published:** 2026-07-20

**Authors:** Vishal A Shetty, Christina M Gregor, Lorraine D Tusing, Eric A Wright, Airín D Martínez, Brendan T O'Connor, David L Chin

**Affiliations:** 1 Center for Pharmacy Innovation and Outcomes, Geisinger, 100 N Academy Ave, Danville, PA, 17822, United States, 1 413-230-4015; 2 Department of Health Promotion and Policy, University of Massachusetts Amherst, Amherst, MA, United States; 3 Department of Investigational Medicine, Geisinger College of Health Sciences, Danville, PA, United States; 4 Department of Pharmacy, Geisinger College of Health Sciences, Danville, PA, United States; 5 Manning College of Information and Computer Sciences, University of Massachusetts Amherst, Amherst, MA, United States

**Keywords:** empathy, communication, patient portals, secure messaging, internet communication, primary health care

## Abstract

**Background:**

Empathic communication in the clinical setting has been associated with improved clinical outcomes, decreased anxiety, and increased patient satisfaction, treatment adherence, and trust. Despite the recent growth in patient portal use, the expression of empathic communication through patient portal secure messages is not well understood.

**Objective:**

This study aimed to construct a coding schema to define and assess empathic opportunities initiated by patients and primary care clinicians’ responses to these opportunities within a patient portal messaging environment.

**Methods:**

Data for this study included adult patient secure messages and responding messages from clinicians working in family practice clinics within a regional health care system serving central and northeast Pennsylvania between January 2018 and December 2023. We conducted a manual review of messages using the Empathic Communication Coding System as a guiding framework, which defines empathic opportunities created by patients and corresponding empathic responses by clinicians. We double-coded 500 patient messages for 3 empathic opportunity types: statements of emotion, progress, and challenge. Coding definitions were iteratively updated to describe specific textual cues unique to the patient portal context. This procedure was repeated to code for empathy in clinician responses to empathic opportunities. An additional 100 patient messages were double-coded for interrater reliability testing, and 500 patient messages were single-coded using the finalized coding schema.

**Results:**

Among 1100 patient messages coded, 576 (52.4%) included an empathic opportunity. Of these messages, 100 (17.3%) included a statement of emotion, 85 (14.7%) included a statement of progress, and 539 (93.6%) included a statement of challenge. Statements of challenge were primarily characterized by patients explicitly describing physical or mental health issues, a barrier in care, or difficulties in their personal lives. Among the 576 patient messages with empathic opportunities, 446 (77.4%) received at least 1 response from a clinician. Clinicians sent 483 response messages, of which 64 (13.2%) expressed empathy.

**Conclusions:**

While patients created empathic opportunities in over half of patient portal secure messages, primary care clinicians infrequently responded with empathy. The findings of this study provide initial evidence of gaps in empathic communication within patient portal secure messages and lay the groundwork for using artificial intelligence models to systematically measure and improve this communication across the patient portal messaging system.

## Introduction

High-quality patient-clinician communication is integral to patient care [1,2] and depends on both the content and the manner in which information is communicated to the patient [3]. A crucial component of effective patient-clinician communication is empathy—the clinician’s ability to recognize and appreciate the patient’s emotions while conveying an understanding of the patient’s lived experience [4]. Empathic communication has been associated with improved clinical outcomes [5] and decreased patient anxiety [6]. Additionally, empathic communication may enhance affective experiences for patients, such as feelings of being listened to, valued, understood, cared for, and validated [7,8]; this may improve outcomes such as satisfaction, treatment adherence, and trust [8]. Empathic communication is viewed as a learnable skill, and its development has been emphasized in health care professional education and training [4,9,10]. Given its importance in the clinical setting, prior studies have made efforts to assess empathic communication [11]. However, these methods are designed around the context of the in-person clinical encounter [12]. This poses a barrier to understanding whether clinicians are communicating empathically with patients over emerging interaction mediums, such as secure electronic messaging within patient portal systems.

Since the passage of the Health Information Technology for Economic and Clinical Health Act in 2009, the adoption and implementation of patient portal systems in the United States have increased considerably [13]. Large multispecialty health systems have seen gradual growth in the volume of secure messages sent by patients, creating a greater responsibility for clinicians to engage with the platform [14,15]. Despite the growth in secure messaging, the expression of empathic communication over this medium is not well understood. Secure messaging allows for a convenient mode of asynchronous communication between patients and clinicians, and while this communication appears to serve a complementary function to in-person encounters rather than a replacement [16], patients are using the platform to initiate questions and discussions about their concerns [17-19].

Prior studies examining communication quality in secure messaging suggest that socioemotional and patient-centered communication may be inconsistently expressed over this medium. In a Veterans Affairs setting, clinicians’ secure messages infrequently included expressions of friendliness, warmth, reassurance, or encouragement [19]. Similarly, analyses of patient portal communication have found that clinician responses to patients’ concerns and requests for assistance often lacked supportive talk or partnership-building language [18]. At the same time, the quality and patient-centeredness of clinicians’ secure messages may influence patients’ perceptions of care and communication quality [20]. These findings suggest that there may be gaps in clinicians’ empathic communication over secure messages, despite its importance to patients. Understanding how clinicians use secure messaging to communicate with patients when presented with the opportunity to express empathy can support the development of tools to measure empathic communication across a patient portal system and can inform interventions aimed at improving clinician communication that responds to patients’ social and emotional needs.

In this study, we aimed to assess empathic communication over patient portal secure message interactions between patients and primary care clinicians. We adapted the Empathic Communication Coding System (ECCS) [21], an established instrument used to identify empathic opportunities expressed by patients and corresponding empathic responses by clinicians during in-person clinical encounters, to the context of patient-clinician electronic text communication. Using this adapted framework, we coded patient and clinician messages for empathic communication and explored how patients create opportunities for clinician empathy and how clinicians respond to these opportunities.

## Methods

### Study Population and Dataset

This study was conducted at Geisinger, a large integrated health system in the United States serving approximately 1 million residents across 45 counties in central and northeastern Pennsylvania [22]. The study population included adult patients (aged ≥18 y) who sent a “Patient Medical Advice Request” secure message via the patient portal to a primary care physician practicing within a Geisinger family practice clinic between January 2018 and December 2023, as well as all clinicians who sent a response message. The “Patient Medical Advice Request” message type is selected by the patient when initiating a secure message and is primarily used when the patient expresses a concern, question, or update related to their care. Patient messages are directed to individual clinicians, but multiple clinicians or office staff may be granted access to view and respond to messages. This category of messages captures the communication between patients and clinicians on this platform. Messages were retrieved by a Geisinger data broker from the system’s clinical data warehouse based on the inclusion and exclusion criteria. Data for this study included 2,232,943 messages sent by 188,828 patients and 1,637,086 responding messages from 4152 clinicians.

### Measuring Empathic Communication

The ECCS was used as a guiding framework to define empathic communication in patient-clinician interactions and to code messages [21]. The ECCS is a validated, reliable instrument that has been used in diverse clinical settings to measure empathic communication with patients and caregivers [23-26]. Although the ECCS was designed in the context of face-to-face interactions, it has been applied effectively to text-only communication between virtual patients and clinicians [27].

The ECCS specifies explicit cues for empathic opportunities—expressions by patients that create windows of opportunity for a clinician to communicate with an empathic response. We coded patient messages for 3 empathic opportunity types defined by the ECCS, which include statements of emotion, progress, or challenge. A statement of emotion occurs when a patient explicitly expresses an emotion or affective state they are experiencing. We additionally distinguished between statements of emotion that were negatively valenced (eg, fear, anger, and sadness) versus positively valenced (eg, happiness, excitement, and relief). A statement of progress occurs when a patient describes a positive development in their physical condition, a psychosocial aspect of their life, or a positive, life-changing event. A statement of challenge, unlike a statement of emotion, does not require any expression of affect but instead occurs when a patient describes a physical or psychosocial problem influencing their quality of life, or a devastating, life-changing event. Multiple types of empathic opportunities may be expressed by a patient in a single message.

If a patient presents an empathic opportunity, a clinician can then respond empathically. The ECCS defines 6 mutually exclusive levels of empathic responses. However, the perfunctory recognition of the patient perspective level is highly nonverbal (eg, an automatic response to a patient while preoccupied with another task, facing away from the patient) and is not applicable to the text-only context of patient portal messages. Therefore, we removed it from the initial coding schema, leaving five levels: (1) denial of the patient perspective, in which the clinician ignores or makes a disconfirming statement to the empathic opportunity; (2) implicit recognition of the patient perspective, in which the clinician focuses on a peripheral aspect of the patient’s statement rather than the empathic opportunity directly; (3) acknowledgment, in which the empathic opportunity is directly addressed; (4) confirmation, in which the clinician conveys to the patient that the empathic opportunity is legitimate; and (5) statement of shared feeling or experience.

### Message Selection for Coding and Schema Adaptation

We selected 500 patient messages for the schema adaptation process using a disproportionate stratified sampling approach, stratifying by patient race and Medicaid status, as race and socioeconomic status may affect language proficiency and expression [28,29]. A sample size of 500 was chosen to balance feasibility with sufficiency to approach concept saturation. Patients with missing data on race or insurance status were excluded from the sampling frame. The eligible dataset included 74.8% (1,640,823/2,193,614) of messages from White non-Medicaid patients, 18.3% (400,456/2,193,614) of messages from White Medicaid patients, 3.9% (84,649/2,193,614) of messages from non-White non-Medicaid patients, and 3.0% (66,484/2,193,614) of messages from non-White Medicaid patients. White patients did not include those of Hispanic or Latino ethnicity. Non-White patients included Black (Black or African American race and not of Hispanic or Latino ethnicity), Hispanic (Hispanic or Latino ethnicity regardless of race), and other (Asian, American Indian or Alaskan Native, Native Hawaiian or other Pacific Islander, 2 or more races, or other race, and not of Hispanic or Latino ethnicity). To increase the representation of messages from non-White patients for the schema adaptation, we oversampled the 2 non-White patient message strata; specifically, each non-White stratum (Medicaid and non-Medicaid) was oversampled to constitute 10% (n=50 each) of the final sample [30]. In oversampling non-White patient messages, we sought to maintain the relative proportion of White Medicaid patients’ messages close to its original proportion in the population. After establishing the adjusted sampling proportions for each stratum, random sampling within each stratum was performed accordingly to reach a total sample size of 500 messages. The final sample composition was as follows: 310 (62.0%) messages from White non-Medicaid patients, 90 (18.0%) messages from White Medicaid patients, 50 (10.0%) messages from non-White non-Medicaid patients, and 50 (10.0%) messages from non-White Medicaid patients. We considered an empathic interaction to be any patient message with an empathic opportunity and the direct response from a clinician. Therefore, clinician messages used in this study’s analysis were selected after patient messages had been analyzed and coded.

### Coding and Schema Adaptation

Before coding messages, 3 study team members (VAS, CMG, and LDT) reviewed literature in which the ECCS was applied in various communication contexts to understand previous approaches used to interpret and adapt the schema definitions [23-25,27,31-38]. The coding process occurred in 3 sequential stages. First, 500 patient messages and corresponding clinician responses were independently double-coded to iteratively adapt the ECCS coding schema to the patient portal messaging context. Second, 100 additional patient messages and corresponding clinician responses were independently double-coded to assess interrater reliability of the finalized coding schema. Third, after sufficient interrater reliability was achieved, an additional 500 patient messages and corresponding clinician responses were single-coded using the finalized schema to increase the number of coded messages included in the primary analysis.

**Figure 1.**
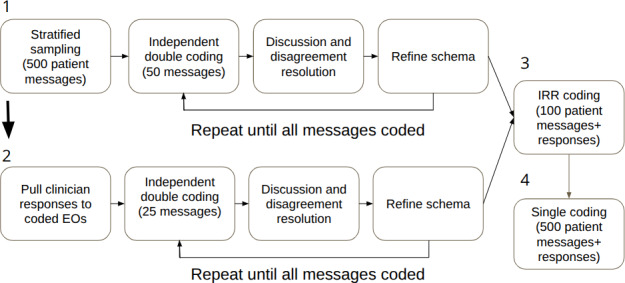
Flow diagram representing the message coding and schema adaptation process. EO: empathic opportunity; IRR: interrater reliability.

The sampled patient messages were independently double-coded by 2 study members (VAS and CMG) for the expression of an empathic opportunity, the types of opportunities present, and the emotional valence if a statement of emotion was present. We intended to code for the intensity or magnitude of the empathic opportunity, but determined it was too difficult to judge from the text alone, given the complexity of the situations described by patients. For the initial round of coding, 50 patient messages were reviewed using the basic definitions of empathic opportunities from the ECCS (Multimedia Appendix 1). Discrepancies in codes were resolved, and suggestions to modify the schema were discussed among the 2 coders and a third member (LDT), after which the coding schema was updated to include any new decision criteria used to determine empathic opportunity types. We repeated this process with 50 messages coded per round until all 500 sampled messages had been coded. A visualization of the coding and adaptation process is presented in Figure 1.

Clinician messages that directly responded to patient messages identified with an empathic opportunity were independently double-coded for empathic response level. Similar to the patient messages, discrepancies in clinician message coding were resolved, and the coding schema was updated with new decision criteria after each round of coding. Given that clinician messages required reviewing subsequent patient messages for the context of the response, 25 messages were reviewed per round of coding (Multimedia Appendix 2). Since a patient message may have more than 1 empathic opportunity statement, we coded clinician messages based on whether the response was directed to at least 1 empathic opportunity statement. For analyses of clinician messages, confirmation and shared feelings or experiences were considered expressions of high empathy. During clinician message coding, we observed responses that were intended to inform the patient that their message had been forwarded to a clinician or that the clinician was currently out of the office. These forwarded messages were not evaluated as empathic responses. Instead, they were identified separately and reviewed to determine whether a subsequent clinician response addressing the original patient message occurred later within the message thread. Any identified follow-up clinician messages were treated as responses to the original patient message and were added to the coded set.

Once the empathic opportunity and response-adapted coding schemas had been finalized, we tested each for interrater reliability (Cohen κ) [39] of at least 0.75 [40] on 100 patient messages and corresponding clinician responses selected using the sampling strategy described earlier. If this threshold had not been achieved, discordant coding decisions would have been reviewed by the study team, the coding schema further refined as needed, and additional batches of messages independently coded until acceptable agreement was achieved. With sufficient interrater reliability achieved, an additional 500 patient messages were selected using our stratified sampling approach and were divided evenly among the 2 coders for single coding of empathic opportunities and corresponding empathic responses.

### Additional Analysis

Empathic opportunity category frequencies were calculated for coded patient messages, and empathic response level frequencies were calculated for coded clinician responses. We conducted a follow-up analysis to investigate why some patient empathic opportunities received no direct response from a clinician within the same message thread. After coding was completed, we identified patient messages containing empathic opportunities without a direct clinician response and randomly sampled 30 messages for detailed review, balancing feasibility with sufficiency to identify common patterns contributing to nonresponse. For each sampled message, we reviewed the patient’s electronic health record activity within 48 hours after the message was sent, including message history, telephone encounters, appointment activity, and orders or prescriptions that may have addressed the patient’s concern outside the original message thread. We reviewed these records to identify contextual factors that may explain the apparent nonresponse.

### Ethical Considerations

This study was approved by the Geisinger Institutional Review Board (IRB00008345). All procedures were performed in accordance with the relevant guidelines and regulations. The study involved secondary research using identifiable patient and clinician data extracted from the Geisinger electronic health record and patient portal system. Access to identifiable data was restricted to approved study team members, and the data were stored within secure Health Insurance Portability and Accountability Act (HIPAA)-compliant Geisinger research infrastructure. No personal identifiers were included in the reported results or study materials.

## Results

### Patient Message Characteristics

A Cohen κ of 0.77 was achieved for empathic opportunity coding. Among the 1100 coded messages (500 for schema adaptation, 100 for reliability test, and 500 for single coding), 52.4% (576/1100) contained an empathic opportunity (Table 1). Among messages with an empathic opportunity, statements of challenge were the most common type expressed (539/576, 93.6%), followed by statements of emotion (100/576, 17.3%; 86/576, 14.9% with a negative emotion and 20/576, 3.5% with a positive emotion) and statements of progress (85/576, 14.7%). In 23.1% (133/576) of messages with an empathic opportunity, more than one type was expressed by the patient.

**Table 1.** Demographic characteristics and their frequencies in patient messages.

Patient characteristic	All patient messages (n=2,232,943)	Coded patient messages (n=1100)	Messages with an empathic opportunity (n=576)
Age^a^ (y), median (SD)	53 (16.7)	47 (17)	46 (16.2)
Insurance status, n (%)			
Medicaid	474,880 (21.3)	308 (28)	166 (28.8)
Non-Medicaid	1,758,063 (78.7)	792 (72)	410 (71.2)
Race or ethnicity^a^, n (%)			
White non-Hispanic	2,041,179 (91.4)	880 (80)	470 (81.6)
Black non-Hispanic	48,883 (2.2)	59 (5.4)	29 (5)
Other non-Hispanic	39,891 (1.8)	40 (3.6)	15 (2.6)
Hispanic	69,507 (3.1)	121 (11)	62 (10.8)
Sex, n (%)			
Female	1,486,377 (66.6)	726 (66)	388 (67.4)
Male	746,566 (33.4)	374 (34)	188 (32.6)

a Patient characteristics did not sum to 100% due to missing data.

### Empathic Opportunities

In the patient portal messaging context, statements of emotion and empathic opportunities were primarily expressed with a negative valence, and patients most frequently explicitly described fears, worry, or concern (Table 2 and Multimedia Appendix 3). In addition to stating emotions directly, patients leveraged unique features of text to indicate their emotions. For example, while describing the challenges they were dealing with, some patients wrote sentences in all capital letters or ended them with an exclamation mark. Patients also used the term “ugh,” and we considered these unique uses of text to be expressions of anger or frustration, as they appear to be intended to convey the patient’s negative feelings.

**Table 2.** Summary of patient portal message–adapted empathic opportunity definitions.

Empathic opportunity category	Summary of category definitions	Examples from data
Statement of emotion	The patient explicitly states or describes feeling an emotion of positive or negative affect. Negative emotions include: Fear Anger or frustration (includes statements with exclamation marks or capitalized words) Hate or displeasure Sadness Shame or guilt Disgust Confusion Concern or worry Desperation Discouragement Positive emotions include: Happiness Excitement Relief	“The reason it slowed down I didnt take the Eliquis for 2 days, I got scared and started taking it again.” “The pill that I tried (i think it was a blood pressure pill) to try to help me get warm did absolutely nothing! Im freezing!” “I cant wait till I get my machine and start feeling better.”
Statement of progress	The patient states or describes a positive development in their physical health and/or mental health, or describes a recent positive, life-changing event. Positive developments include: Improved health condition Improved health behavior Improved care experience Overcoming obstacles Milestone changes in personal life	“The one to claim me down and help with Anxieties and Seizures. It has been doing a great job I need a refill of it.” “At least I’m not sick anymore lol.”
Statement of challenge	The patient states or describes a physical, mental, or psychosocial condition or issue that could negatively impact the quality of their life, or describes a recent negative, life-changing event. Challenges include: Physical health symptom or issue Mental health symptom or issue General difficulty Ineffective treatment Care discomfort Barrier in care Financial struggles Transportation issues Personal life-changing event	“I’m still feeling horrible. Yesterday I was throwing up & today I have the diarrhea. All I’m doing is sleeping & I go from being really hot to having the chills.Im hoping to feel better soon.” “The betamethasone cream doesn’t work for me. I’d like to have the clobetasol propionate cream.” “Can you send one more refill? Im not sure if I had a UTI or not, nobody ever called me to let me know what was going on.”

Patients expressed statements of progress and empathic opportunities primarily to convey an improvement in their condition. This included patients providing a general update that they were recovering well or feeling better, or stating that a particular medication or treatment had worked for them. Though less frequent than reporting condition improvements, patients also described improvements in their care experience, such as feeling listened to.

The statement of challenge empathic opportunities was primarily characterized by patients explicitly describing physical or mental health issues, a barrier in care, or difficulties in their personal lives. While patients often directly described recent experiences with physical and mental health issues such as pain, injury, fatigue, stress, and anxiety, they also alluded to these issues by describing abnormal test results, exposure to a person-transmissible illness (often COVID-19 in these data), or a surgery that had occurred or was scheduled. Patients also expressed nonhealth challenges related to barriers in care, such as perceived delays in being seen, lack of follow-up communication, difficulties scheduling an appointment or accessing a clinician, or medication being sent to the incorrect pharmacy. Significant personal challenges conveyed by patients included dealing with the death or end-of-life process of a family member or friend, losing employment, or having been in prison.

### Clinician Message Characteristics

A Cohen κ of 0.91 was achieved for empathic response level coding. Clinicians sent 483 response messages to 446 patient messages containing an empathic opportunity (Table 3). The coded response messages were sent by at least 276 unique clinicians. This count does not include messages sent from pooled physician office accounts, to which no single clinician could be attributed. Therefore, the total number of individuals represented in the coded response set was likely higher. The majority of responses to empathic opportunities were acknowledgment (328/483, 67.9%). A confirmation response was expressed in 13% (63/483) of clinician messages, and an implicit recognition of the patient perspective occurred in 10.8% (52/483). Denial of the patient perspective and a shared feeling or experience response occurred infrequently (5/483, 1% and 1/483, 0.2%, respectively). We identified 7% (34/483) of responses as forwarded messages. For 47.1% (16/34) of these forwarded messages, a clinician later responded directly to the original patient message (these responses were included in the coded set). For the remaining 52.9% (18/34) of these messages, 10 messages received a response from a clinician as a reply to the forwarded message. These clinician messages included 7 acknowledgment responses and 3 confirmation responses.

**Table 3.** Demographic characteristics and clinician type frequencies in response messages.

Clinician characteristic	All clinician response messages (n=1,637,086)	Coded clinician response messages (n=483)	Response messages with high empathy^a^ (n=64)
Age (y), median (SD)	43 (11.9)	42 (12.2)	39.5 (10.2)
Race or ethnicity^b^, n (%)			
White non-Hispanic	1,406,107 (85.9)	393 (81.4)	46 (71.9)
Black non-Hispanic	21,953 (1.3)	15 (3.1)	3 (4.7)
Asian	141,753 (8.7)	57 (11.8)	13 (20.3)
Hispanic	23,474 (1.4)	6 (1.2)	2 (3.1)
Two or more	16,729 (1.0)	6 (1.2)	0 (0)
Other non-Hispanic	323 (0.02)	0 (0)	0 (0)
Sex^b^, n (%)			
Female	1,113,159 (68.0)	326 (67.5)	38 (59.4)
Male	508,020 (31.0)	156 (32.3)	26 (40.6)
Clinician type, n (%)			
Nurses^c^	175,799 (10.7)	53 (10.9)	5 (7.8)
Physicians	938,053 (57.3)	293 (60.6)	49 (76.6)
Physician assistants	43,492 (2.7)	16 (3.3)	1 (1.6)
Physician’s office^d^	465,334 (28.4)	121 (25.1)	9 (14.1)

a High empathy includes “Confirmation” and “Shared Feeling or Experience” responses.

b Clinician characteristics did not sum to 100% due to missing data.

c Includes licensed practical nurses, registered nurses, certified registered nurse practitioners, and nursing assistants.

d A pooled account that includes nurses, medical assistants, and office staff represents the primary care physician to whom the patient message was sent.

### Empathic Responses

Clinician denial of the patient perspective and shared feeling or experience responses were infrequently observed (n=5 and n=1, respectively), and category definitions for these response levels did not differ substantially between the initial coding schema and the final schema adapted to the context of patient portal communication (Table 4 and Multimedia Appendix 4). Clinician implicit recognition of the patient perspective responses was primarily characterized by deferring discussion of the empathic opportunity to a future date. Instead of using the patient portal to at least acknowledge the empathic opportunity statement, some clinicians would tell patients to schedule an appointment or come to a clinic, even if the patient did not ask for an appointment. While such responses did not outright ignore the perspective of patients, this communication failed to provide a clear indication that the empathic opportunity had been recognized and understood.

**Table 4.** Summary of patient portal message–adapted empathic response definitions.

Empathic response category	Summary of category definitions	Examples from messages
Denial of patient perspective	The clinician ignores or makes a disconfirming statement in response to the patient’s empathic opportunity. This may include: Immediately changing topic Ignoring the empathic opportunity Trying to invalidate the patient’s empathic opportunity	Patient: “Are eggs safe with high cholesterol I researched And it says that it’s OK and then it says it’s not OK Hey I just wanna make sure.” Clinician: “Order was placed for GI.”
Implicit recognition of patient perspective	The clinician responds to the patient, but not directly to the empathic opportunity statement. This may include: Recommending to schedule an appointment without addressing empathic opportunity Deferring addressing empathic opportunity to a future date Not clearly or directly addressing empathic opportunity	Patient: “Sorry to be a pain but it’s been months of this pain on my right side and it’s becoming unbearable. Yesterday was one of the bad days where it hurt to even take a deep breathe. Did my MRI show anything? If not, is there anything else to be looked at? This can’t just be the way it is.” Clinician: “Come in for an appointment in person. Please call to schedule.”
Acknowledgment	The clinician acknowledges the patient’s empathic opportunity statement directly. This may include: Restating the empathic opportunity Providing direct guidance or follow-up questions to the empathic opportunity Responding to an inquiry that is a direct consequence of the empathic opportunity	Patient: “The cyst still hurts, and I’m scheduled this [DAY] for removal. Should the antibiotics and/or the [DAY]’s date be changed?” Clinician: “Does it look less inflamed, tender? If so we can stop the antibiotics and wait until seen by surgery. If not I can switch to a different antibiotic to see if that’s more tolerable.”
Confirmation	The clinician conveys to the patient that the expressed empathic opportunity statement is legitimate. This may include: Providing affirming statements Expression of concern or sympathy Providing reassurance Apologizing when patient perceives clinician’s fault Wishing the patient well Sharing the experience of other patients	Patient: “You had given me a script for my groin pain, but I didn’t keep going as it seems better. Then I tore my meniscus, and have an appt with a surgeon to discuss surgery. Then I tripped and came down hard on my foot which caused a pull in the tendon or ligament in my hip-butt area. I know my Sjögren affects connective tissues. So can you send a new script to [CLINIC] for the multiple issues?” Clinician: “That’s a lot going on, so sorry to hear that. We’ll fax over a referral and you can give them a call. I hope you feel better.”
Shared feeling or experience	The clinician makes an explicit statement that he or she shares the patient’s emotion or has had a similar experience as the patient. This may include: Expressing the same emotion as the patient Sharing a similar experience as the patient	Patient: “The specialist from [CLINIC] said the blood issue is not blood disease. He did not have a clear reason for why this is happening. Hypoxia is his guess. That is a bit confusing because I am on CPAP with 2 liter oxygen and all pulmonary test appear normal. At this point [CLINIC] is not in the picture for continued treatment do you have any ideas of where I go next? Clinician: “I wouldn’t go anywhere else at this point except to follow over time and watch. Don’t think anything else will be done except for phlebotomy, which is sometimes the treatment for secondary polycythemia. Frustrating that a place like [CLINIC] did not give better follow-up instruction.”

The most common response clinicians made was an acknowledgment of the empathic opportunity. These responses usually reflected the clinician’s routine clinical role—directly addressing the patient’s expressed challenges, progress, or emotions. This often included offering guidance, clarifying uncertainties, or posing follow-up questions while referencing the specific health issue or concern raised. In some cases, patients made requests that followed directly from an empathic opportunity (eg, asking for a prescription after describing a challenge or progress), and clinicians would only respond to the inquiry without explicitly acknowledging the empathic opportunity statement. We considered these responses to be acknowledgment, as directly addressing the inquiry effectively served to address the empathic opportunity.

Confirmation responses were characterized by clinicians communicating in a manner that legitimized or affirmed the patient’s emotions, progress, or challenges, going beyond addressing the empathic opportunity with clinical or procedural information. This often entailed clear and simple statements of affirmation or consolation, such as “I’m glad to hear that is working for you” in response to a statement of progress, or “I’m sorry you have to deal with that” in response to a statement of challenge. In situations where the patient expressed a statement of emotion or challenge due to a perceived fault with the clinician, some responses included apologies, even if the clinician may not have been at fault, which we considered to be empathic (eg, “I apologize that you haven’t received a message back yet”).

### Nonresponses From Clinicians

We found that 22.6% (130/576) of patient empathic opportunities did not receive a direct clinician response within the same message thread. From the random sample of these messages, we constructed 4 broad groups. For 30% (9/30) of messages, clinicians likely perceived that no response was necessary due to the way the patient framed their communication, which often consisted of updates on planned next steps or restatements of previously resolved concerns. For 23.3% (7/30) of messages, while a response message was not provided, clinicians took subsequent action related to the empathic opportunity statement, such as calling the patient, refilling a prescription, ordering a test, or scheduling an appointment. For 20% (6/30) of messages, clinicians responded to the empathic opportunity in a different thread, primarily because the patient initiated multiple message threads about the same issue over a short period of time. For the remaining 26.7% (8/30) of messages, no additional context could be determined from the patient record, indicating that the messages were either ignored or no documentation was made related to follow-up activity.

## Discussion

### Principal Findings

This study represents the first investigation into the expression of patient empathic opportunities and corresponding clinician responses in the context of patient portal secure messages. Among 1100 patient messages analyzed, we found that 576 (52.4%) contained at least 1 empathic opportunity. The majority of empathic opportunities expressed were statements of challenge, primarily characterized by descriptions of physical or mental health issues and barriers to accessing care. Of the patient messages with an empathic opportunity, 446 (77.4%) received a response message from a clinician. Clinicians sent 483 response messages, of which 64 (13.2%) contained expressions of empathy. Most clinician responses were acknowledgments of an empathic opportunity in which the patient’s statement was directly addressed, but the response did not provide more than clinical or procedural information.

The prevalence of empathic opportunities we observed in patient portal messages aligns with how patients typically use the platform to communicate with clinicians. Previous studies have explored the primary topics discussed in patient portal messages, and while common topics include those in which empathic opportunities may not be present (eg, prescription requests and appointment scheduling), many topics are likely to be headlined by an empathic opportunity statement (eg, discussions of symptoms, treatment planning, status updates, and administrative or logistical issues) [17,41-43]. It should be noted that the type of message analyzed in this study is intended to be selected by the patient when they are seeking medical advice (although our observations indicated patients sometimes used them for other purposes), which may contribute to the prevalence of empathic opportunities. Additionally, the primary care setting of this study may also explain why the overwhelming majority of empathic opportunities expressed were statements of challenge. Applications of the ECCS in specialty settings have yielded other distributions of empathic opportunity types [23,24]. For example, statements of emotion were the most common empathic opportunity type expressed by patients with lung cancer [23] and caregivers of hospice patients [24], which is likely related to the severity of illness involved in these settings [44,45]. Patient messages in this study were directed to family practice clinics, where the range and severity of issues discussed are diverse [46]. Further research is needed to understand if and how patients express empathic opportunities in messages differently based on clinical setting. Understanding these differences could assist clinicians in anticipating and effectively responding to the types of empathic opportunities most commonly encountered.

While clinicians generally appreciate the efficiency and convenience afforded by patient portal messaging [47-50], some have reported frustration with aspects of communication on the platform, including patients’ inappropriate and informal messaging habits, prompting clinicians to carefully consider when and how they respond to avoid encouraging excessive messaging [47-49]. This may partially explain our finding that most clinicians responded to patients by acknowledging empathic opportunities—a response characterized by directly addressing the patient’s statement without further legitimizing or validating the patient’s experience. Communication over secure messages has tended to be informational and transactional in nature [51,52]. Analyses of clinician secure messages have found that information-giving is the predominant form of patient-centered communication, while supportive talk, partnership-building language, reassurance, and expressions of warmth occur less frequently [18,19,51]. Other studies have also observed variability in how clinicians approach emotional content in secure messaging contexts [53]. Messaging over the patient portal may be shaped by norms and workflows that prioritize efficient information exchange and task completion, while empathic and patient-centered communication behaviors may be prioritized less consistently.

Similar to our findings, in prior evaluations of general internists during in-person encounters, acknowledgment emerged as the most common response to empathic opportunities [21,25]. This suggests that acknowledgment may represent a standard response to empathic opportunities for many primary care clinicians, regardless of the communication platform. This type of direct response may be sufficient for patients with simple requests or questions [20,47,48,54]. However, clinician messages that convey a more socioemotional tone can help strengthen rapport with patients [55], and the quality of communication may affect patient perceptions of care [56], which may indicate the importance of incorporating empathic language into clinician responses. We found that responses reflecting higher levels of empathy, such as confirmation or shared feeling, occurred relatively infrequently, despite often requiring only brief expressions of validation or emotional recognition. This suggests that the rarity of higher-level empathic responses may not be explained solely by clinicians prioritizing efficient, goal-directed communication. Rather, these findings raise questions about how clinicians conceptualize empathic communication in asynchronous settings and whether existing communication norms adequately support the expression of empathy in patient portal interactions. Future work should examine clinician perspectives regarding empathic communication in digital care settings and identify factors that influence when and how empathic responses are expressed.

In the original ECCS framework, the lowest level of empathic response, denial of patient perspective, includes ignoring an empathic opportunity. This classification is appropriate in the context of an in-person encounter, where such inaction is difficult to interpret as anything other than a dismissal of the patient’s statement. We initially considered categorizing clinician nonresponses as denials of the patient perspective but reconsidered after examining the context. While some empathic opportunities did not appear to be addressed, most nonresponses did not suggest intentional neglect by the clinician. Although prior studies have found that clinicians rarely fail to recognize these patient cues during in-person encounters [26], our findings diverge. We observed a higher prevalence of nonresponses and messages that appear dismissive (ie, implicit recognition of the patient perspective), which may reflect communication norms specific to the patient portal. These patterns are likely influenced by the burden of message volume, a known contributor to burnout and fatigue previously reported in several different clinical settings [48,57,58]. Automated methods for triaging messages [59-61] and programs introducing message billing [62] are being explored to reduce message volume and clinician burden, and the implementation of such solutions may be necessary before considering interventions to improve clinician responsiveness to empathic opportunities.

While this study provides insight into how patients express empathic opportunities through patient portal messages and highlights potential gaps in clinician responses, system-wide identification of empathic communication in messages would enable a more comprehensive analysis of communication patterns. This includes examining patient characteristics associated with empathic opportunities, differences in empathic responsiveness across clinician subgroups, and changes in empathic communication over time. Previous studies have attempted to use natural language processing (NLP) methods to measure empathy in textual dialog, but they use conceptualizations of empathy specific to synchronous communication, nonclinical dialog, and mental health counseling contexts, which may not be applicable to the patient portal messaging context [63]. Our work adapts a framework for patient-clinician interactions [21] to build a set of ontologies for categorizing the expression of empathic opportunities and responses in patient portal messages. These ontologies may serve as the foundation for developing NLP models to recognize empathic communication. Large language models have shown promise for text classification tasks in health care settings, though applications exploring patient portal messages are relatively sparse [64]. Further research is needed to determine how large language models may be effectively applied to identify empathic opportunities and responses in messages to enable large-scale analysis.

### Limitations

Our study has several limitations. First, while our disproportionate stratified sampling approach accounted for patient race and Medicaid status, we did not consider clinician characteristics, which may affect the frequency of empathy we observed. Clinician messages included in this study’s analysis were dependent on the patient messages sampled, as we only coded responses to patient messages with an empathic opportunity. However, patient-clinician shared characteristics, such as racial and gender concordance, have been found to be associated with higher communication quality [65,66]. Any large-scale analysis of empathic communication over patient portal messages should account for differences in empathic responses in interactions where patient and clinician characteristics are concordant versus discordant. Second, we were unable to account for factors external to the message interaction context. For example, existing patient-clinician rapport, information exchanged during previous in-person visits, and clinician workload may affect the expression of empathy. Finally, our adaptation of the ECCS to the patient portal context may not be generalizable across clinical settings, as messages included in this study were sent to and from family practice clinics within one health system. Our determination of empathic response level definitions was based on our interpretation of the ECCS in this asynchronous primary care interaction context and our observations of message content from this specific patient and clinician population. For example, we considered simple statements of affirmation as empathic (ie, confirmation response level) in part because these responses were not necessary to address the often direct and transitory patient inquiries expressed in this setting, but they nevertheless represented a degree of attentiveness to the patient’s empathic opportunity above acknowledgment. However, strict criteria for empathy may be warranted in other contexts of patient portal communication. In mental health care contexts, the standard for empathy may be higher than in primary care, requiring clinicians to probe for the patient’s story through question-asking and reflective listening techniques in order to adequately address patient concerns [67]. Further research is needed to understand how patient portal communication differs depending on the clinical setting and patient population, and whether these differences change the kind of response that is considered empathic.

### Conclusions

We adapted an established framework for measuring empathic communication during in-person encounters to the patient portal messaging context and described how primary care clinicians respond to empathic opportunities created by patients over this communication platform. Patients primarily created opportunities for empathy by describing personal challenges. While clinicians infrequently expressed empathy explicitly in their responses, the majority acknowledged and directly addressed a patient’s empathic opportunity statement. The ability to convey empathy through asynchronous communication may be critical to maintaining care quality outside of in-person encounters. Health systems may prioritize reducing the burden of high message volume experienced by clinicians to facilitate improved empathic communication. Future research is needed to develop scalable methods for a systematic understanding of patterns and gaps in clinician empathy. The ontology for empathic communication presented in our study may provide a foundation for training NLP models to support large-scale analyses.

## Supplementary material

10.2196/87195 Multimedia Appendix 1 Initial empathic opportunity coding guidelines.

10.2196/87195 Multimedia Appendix 2 Initial empathic response coding guidelines.

10.2196/87195 Multimedia Appendix 3 Final empathic opportunity coding guidelines.

10.2196/87195 Multimedia Appendix 4 Final empathic response coding guidelines.

## Abbreviations

ECCS: Empathic Communication Coding System
HIPAA: Health Insurance Portability and Accountability Act
NLP: natural language processing
