# Supplementary material for: Defining and Assessing Empathic Communication in Patient Portal Secure Messages: Adapted Coding Framework Development Study
Source: JMIR Form Res. 2026 Jul 20;10:e87195. doi: 10.2196/87195 (PMC13384347; doi:10.2196/87195)
Supplement: Multimedia Appendix 1 [file formative-v10-e87195-s001.docx]

**Initial empathic opportunity coding guidelines**

For each patient message, coders were asked the following questions:

1. Statement of Emotion: Does the patient explicitly state or describe feeling an emotion of positive or negative affect?

- No (0)
- Yes (1)

1A. Negative Affect: If yes to 1, is an emotion of negative affect stated or described?

- No (0)
- Yes (1)

1B. Positive Affect: If yes to 1, is an emotion of positive affect stated or described?

- No (0)
- Yes (1)

Examples of negative emotions:

- Fear: “I’m scared…”; “My biggest fear is…”

- Anger/frustration: “I’m really mad…”; “I’ve been angry at…”; “I’m frustrated that I can’t…”

- Hate/displeasure: “I really hate...”; “I really don’t like…”

- Sadness: “I’m sad…”; “I’ve been pretty down lately”; “I’ve been crying/in tears…”

- Shame/Guilt: “I feel ashamed/guilty…”; “I wanted to apologize…”;

Examples of positive emotions:

- Happiness/joy: “I’m happy…”;

- Excitement: “I can’t wait to…”

- Relief: “I’m just relieved…”

2. Statement of Progress: Does the patient (or proxy) state or describe a positive development in their physical health and/or mental health, or describe a recent positive, life-changing event?

- No (0)
- Yes (1)

Examples of positive developments:

- Improved health behavior

→ Patient states that they quit smoking

→ Patient states that they are exercising more

- Improvement in condition

→Patient states that they are recovering well/feeling better

→ Patient states that they no longer have to take medication

- Change in personal life

→Patient states that they recently got married

→Patient states that they recently had a child

3. Statement of Challenge: Does the patient (or proxy) state or describe a physical, mental, or psychosocial condition/illness/issue that could negatively impact the quality of their life, or describe a recent negative, life-changing event?

- No (0)
- Yes (1)

Examples of challenges:

- Any physical health symptom or issue explicitly stated by the patient

→Fever, pain, coughing, infection, headache/migraine

- Any mental health symptom or issue explicitly stated by the patient

→Anxiety, depression, uncontrolled thoughts, manic episodes

- Lack of sleep, fatigue, low energy, tiredness, exhaustion

- Death of someone close to the patient

- Financial struggles
