# Supplementary material for: Defining and Assessing Empathic Communication in Patient Portal Secure Messages: Adapted Coding Framework Development Study
Source: JMIR Form Res. 2026 Jul 20;10:e87195. doi: 10.2196/87195 (PMC13384347; doi:10.2196/87195)
Supplement: Multimedia Appendix 2 [file formative-v10-e87195-s002.docx]

**Initial empathic response coding guidelines**

For each clinician response message, coders were asked the following questions:

For each clinician message, select the response type that best applies (only one may be selected)

1. Denial of patient perspective: Does the clinician ignore or make a disconfirming statement in response to the patient’s empathic opportunity?

- No (0)
- Yes (1)

Examples might include:

- The clinician is making an immediate topic change.

- The clinician is not responding at all

- The clinician making a statement that invalidates or tries to disprove the patient’s statement.

2. Implicit recognition of patient perspective: Does the clinician give a response focusing on a peripheral aspect of the patient’s empathic opportunity, rather than on the central issue?

- No (0)
- Yes (1)

Examples might include:

- The clinician’s statement is more content-based, not dealing directly with the progress, challenge, or emotion.

- The clinician asks questions or gives advice not directly about the empathic opportunity, but about another aspect of what the patient said.

3. Acknowledgement: Does the clinician acknowledge the patient’s empathic opportunity statement directly?

- No (0)
- Yes (1)

Examples might include:

- The clinician paraphrases, summarizes, or restates what the patient said

- Clinician asks a question, makes a statement, or offers advice or help directly about the empathic opportunity

4. Confirmation: Does the clinician convey to the patient that the expressed empathic opportunity statement is legitimate?

- No (0)
- Yes (1)

Examples might include:

- The clinician makes a congratulatory remark to a statement of progress

- The clinician states that the challenge the patient is experiencing is difficult.

- The clinician states that it is understandable for the patient to feel a certain emotion.

- The clinician states that other patients have experienced the same emotion, progress, or challenge.

5. Shared feeling of experience: Does the clinician make an explicit statement that he or she shares the patient’s emotion or has had a similar experience as the patient?

- No (0)
- Yes (1)

Examples might include:

- The clinician uses the phrase, “in my experience”
