# Supplementary material for: Defining and Assessing Empathic Communication in Patient Portal Secure Messages: Adapted Coding Framework Development Study
Source: JMIR Form Res. 2026 Jul 20;10:e87195. doi: 10.2196/87195 (PMC13384347; doi:10.2196/87195)
Supplement: Multimedia Appendix 3 [file formative-v10-e87195-s003.docx]

**Final empathic opportunity coding guidelines**

For each patient message, coders answered the following questions:

1. Statement of Emotion: Does the patient explicitly state or describe feeling an emotion of positive or negative affect?

- No (0)
- Yes (1)

1A. Negative Affect: If yes to 1, is an emotion of negative affect stated or described?

- No (0)
- Yes (1)

1B. Positive Affect: If yes to 1, is an emotion of positive affect stated or described?

- No (0)
- Yes (1)

Examples of negative emotions:

Fear: “I’m scared…”; “My biggest fear is…”

→Includes patient stating they have a phobia

Anger/frustration: “I’m really mad…”; “I’ve been angry at…”; “I’m frustrated that I can’t…”

→ Patient uses an exclamation mark (!) when making a statement of challenge

→ Patient capitalizes key words/phrases that make up a statement of challenge

→ Patient uses the word “Ugh”

Hate/displeasure: “I really hate...”; “I really don’t like…”

Sadness: “I’m sad…”; “I’ve been pretty down lately”; “I’ve been crying/in tears…”

Shame/Guilt: “I feel ashamed/guilty…”; “I wanted to apologize…”

Disgust: “I’m disgusted by...”

Confusion: “I’m so confused...”

Concern/worry: “I’m concerned/worried...”; “I have a concern/worry...”

Desperation: “I desperately need...”

Discouragement: "Its very discouraging"

Examples of positive emotions:

Happiness/joy: “I’m happy…”

Excitement: “I can’t wait to…”

→ Patient uses an exclamation mark (!) when making a statement of progress

→ Patient capitalizes key words/phrases that make up a statement of progress

Relief: “I’m just relieved…”

Does NOT include expressions of thanks

2. Statement of Progress: Does the patient state or describe a positive development in their physical health and/or mental health, or describe a recent positive, life-changing event?

- No (0)
- Yes (1)

Examples of positive developments:

Improved health behavior

→ Patient states that they quit smoking

→ Patient states that they are exercising more

→ Patient states that they are eating healthier

→ Patient states they had a (desired) change in weight

Improvement in condition

→Patient states that they are recovering/feeling better

→ Patient states that they no longer must take a medication

→ Patient states that treatment/medication has been working for them

Improvement in care experience

→Patient states that they feel they are being listened to

Overcoming obstacles

→ Patient describes themselves acting in a courageous/brave way

Milestone changes in life

→Patient states that they recently got married

→Patient states that they recently had a child

→Patient states that they recently found a new job

3. Statement of Challenge: Does the patient state or describe a physical, mental, or psychosocial condition/illness/issue that could negatively impact the quality of their life, or describe a recent negative, life-changing event?

- No (0)
- Yes (1)

Examples of challenges:

Any physical health symptom or issue explicitly stated by the patient

→Fever, pain or physical discomfort, injury, coughing, infection, headache/migraine, lack of sleep, fatigue, low energy/tiredness/exhaustion, nausea, stomach issues, itching, undesired weight change, abnormal test results (from the patient’s perspective), exposure to another person with a transmissible illness, memory loss/difficulty with memory, inflammation, hearing issues

→This includes the patient explicitly stating a negative physical health issue as a reason for requesting a prescription

→This includes the patient expressing their physical health issue through a metaphor, simile, or analogy (e.g., “it feels like there are a hundred needles in my foot”)

→This includes the patient mentioning a surgery they have had or will have.

→This does NOT include a patient indicating that they took or will take a preventive measure for a physical health issue they currently do not have (e.g., a vaccine, checking for abnormalities, etc.)

Any mental health symptom or issue explicitly stated by the patient

→Anxiety, depression, stress, uncontrolled thoughts, manic episodes

→ This includes the patient explicitly citing a negative mental health issue as a reason for requesting a prescription

→This includes the patient expressing their mental health issue through a metaphor, simile, or analogy

General challenge

→Patient makes a general statement about having difficulty, challenge, a tough time, a bad day, etc.

Ineffective treatment

→Patient states that a treatment/medication does/has not worked for them

Functional challenges

→Patient states that they cannot function/do things as they normally do

Care discomfort

→Patient states they are hesitant/uncomfortable with a procedure or treatment

Barrier in care

→Waiting too long/delayed in care (according to the patient)

→Never received a response back (i.e., a call or message),

→Patient states they were ignored, and feel like they are not being heard

→ Unable to view or never received a test result that was expected

→ Forced to see a new clinician

→ Having difficulty scheduling an appointment or seeing/contacting a clinician

→ Having difficulty navigating the patient portal

→ Believes they were incorrectly scheduled for an appointment

→ Insurance not being accepted or the insurer denying coverage

→ Losing insurance

→ Medication was sent to the incorrect pharmacy

→ Medication not available at pharmacy

→ No/limited access to medications the patient perceives they need

→ Error in the medication instruction or listing.

→ This does NOT include typical processes in the delivery of care (i.e., patient states they require a referral, prior authorization)

- Financial struggles

→Patient indicates challenges in affording services/covering costs

→ This includes the patient mentioning receiving forgiveness or assistance for covering the cost of a healthcare service

Transportation

- Patient describes challenges getting to a healthcare facility or pharmacy

Life-changing event

→Patient mentions the death of a family member/friend

→Patient describes hospice admission for a family member/friend

→Patient mentions being fired/losing employment

→Patient mentions being in prison or having significant legal issues
