# Supplementary material for: Defining and Assessing Empathic Communication in Patient Portal Secure Messages: Adapted Coding Framework Development Study
Source: JMIR Form Res. 2026 Jul 20;10:e87195. doi: 10.2196/87195 (PMC13384347; doi:10.2196/87195)
Supplement: Multimedia Appendix 4 [file formative-v10-e87195-s004.docx]

**Final empathic response coding guidelines**

For each clinician response message, coders were asked the following questions:

Select the response type that best applies (only one may be selected)

1. Forwarded message: Does the response indicate that the message will be forwarded to another clinician?

- No (0)
- Yes (1)

Examples include:

→Response states that the message has been forwarded/sent to the clinician

→Response asks if the patient would like the message to be sent to a specific clinician

→Response states that the clinician is away and will receive the message later

→Response does NOT include anything that addresses the patient’s statement

2. Denial of patient perspective: Does the clinician ignore or make a disconfirming statement in response to the patient’s empathic opportunity?

- No (0)
- Yes (1)

Examples include:

- The clinician is making an immediate topic change.

- The clinician is ignoring the empathic opportunity.

- The clinician making a statement that invalidates or tries to disprove the patient’s statement.

Examples include:

→The clinician only responds to an inquiry made by the patient but ignores the empathic opportunity statement.

→ For example, the patient says: “What were the results of the urine test? Also, I wanted to let you know that the pain in my leg is back.” In response, the clinician only addresses the inquiry: “We won’t receive the results until tomorrow”.

3. Implicit recognition of patient perspective: Does the clinician respond to the patient, but not directly to the empathic opportunity statement?

- No (0)
- Yes (1)

Examples include:

Recommendation to schedule an appointment

→In response to a statement of challenge or emotion, the clinician asks the patient to schedule an appointment or points out an upcoming appointment when the patient did not explicitly ask for or mention an appointment.

→In response to a statement of progress in which the patient asks a question directly related to the progress (e.g., asking if they can continue a drug that is working for them), the clinician asks the patient to schedule an appointment, without directly addressing the question.

Deferral to a future date

→In response to a patient request directly related to an empathic opportunity, the clinician responds with “we will see” or “we will discuss another time” without first directly addressing the patient’s statement.

Not directly or clearly addressing the statement

→ In response to a patient’s inquiry with an empathic opportunity statement, the clinician responds in a way that may not clearly answer the patient’s inquiry.

→ For example, the patient says: “Will the pain go away by itself or should anything more be done?” In response, the clinician says: “The fluid is being reabsorbed by your body.”

4. Acknowledgement: Does the clinician acknowledge the patient’s empathic opportunity statement directly?

- No (0)
- Yes (1)

Examples of responses to statements of challenge or emotion include:

Restatement

→ The clinician paraphrases, summarizes, or restates part of the patient’s statement of challenge or emotion

Directly addresses the statement

→The clinician asks a clarifying question, offers advice, or helps directly with the challenge or emotion

→ In response to a patient stating that a treatment isn’t working, the clinician asks if the patient would like to try an alternative

→ In response to a patient describing a barrier in care, the clinician tries to explain the issue, resolves the issue directly, or advises the patient on how to resolve the issue.

→If the clinician cannot address the challenge described by the patient, the clinician explains why

→ In response to the patient expressing confusion or uncertainty in a treatment plan, the clinician attempts to clear up the confusion/uncertainty

→ In response to the patient describing a current or future care plan in which a statement of challenge is mentioned, the clinician gives affirmation to the plan (e.g., “I agree with the plan”; the plan is good; “you are doing the doing right thing”; “Let me know how it goes”).

→ In response to the patient updating their current condition in which a statement of challenge is mentioned, the clinician acknowledges the update directly (e.g., “Thanks for the update”; “continue to keep me posted”).

→In response to the patient requesting an action directly related to a statement of challenge (e.g., an order for an MRI or X-ray, a new prescription or refill, an appointment or referral, documentation), the clinician addresses the request.

→In response to the patient’s statement, the clinician offers that they will/should be able to help the patient’s issues

Example responses to statements of progress include:

→In response to the patient saying they are doing well on a prescription/treatment and makes an inquiry (e.g., asks about next steps), the clinician directly answers the patient's inquiry

5. Confirmation (Empathy): Does the clinician convey to the patient that the expressed empathic opportunity statement is legitimate?

- No (0)
- Yes (1)

Examples include:

Response to statement of emotion

→The clinician states that they understand the emotion the patient is feeling

→The clinician states that other patients have experienced the same emotion in a similar situation

Response to statement of progress

→The clinician makes a congratulatory remark to a statement of progress

→ The clinician shares the experience of other patients who have had similar progress

→ The clinician states they are happy/glad to hear about the patient’s progress

Response to the statement of challenge

→The clinician states that the challenge the patient is experiencing is difficult/a lot to handle.

→The clinician affirms that the challenge the patient is experiencing is concerning

→The clinician reassures the patient not to worry about the challenge they are dealing with

→ The clinician states that they are sorry to hear that the patient is experiencing the challenge

→ The clinician states that they are sorry in response to a challenge which may be perceived as the responsibility of the health system/clinician (e.g., “I’m sorry you didn’t receive a message back...”; “I apologize for the delay in getting your results back”)

→The clinician gives a well-wishing statement (e.g., “hope you feel better/have a quick recovery”; good luck with your treatment.”)

→The clinician makes an affirming statement to the patient (e.g., “That’s a good question; “I respect the decision you’ve made”).

→ The clinician shares the experience of other patients who have had similar challenges

6. Shared feeling or experience: Does the clinician make an explicit statement that he or she shares the patient’s emotion or has had a similar experience as the patient?

- No (0)
- Yes (1)

Examples might include:

→The clinician shares in an emotion the patient is likely feeling (e.g., “It’s frustrating that you had to deal with that”)

→ The clinician shares their own experience of feeling the same emotion, or having a similar progress or challenge (e.g., “I’ve been dealing with pain in my right leg for many years, and it can be very difficult”)
